# Unraveling the complexities of drought stress in cotton: a multifaceted analysis of selection criteria and breeding approaches

**DOI:** 10.7717/peerj.17584

**Published:** 2024-06-24

**Authors:** Hatice Kübra Gören, Uğur Tan

**Affiliations:** Adnan Menderes University, Aydın, Turkey

**Keywords:** Cotton, Selection criteria, Drought stress, Cluster analysis

## Abstract

Abiotic stress tolerance breeding programs present a spectrum of perspectives, yet definitive solutions remain elusive, with each approach carrying its own set of advantages and disadvantages. This study systematically evaluates extant methodologies, comparing plant performance across varied genotypes and selection traits under optimal and stress conditions. The objective is to elucidate prevailing ambiguities. Ten homozygous lines (F8 generation) were assessed using a randomized block design alongside five control varieties, with four replicates cultivated under well-watered and deficit water conditions. It is noteworthy that six of the ten homozygous lines were cultivated exclusively under well-watered conditions (F3 to F7), while four lines experienced deficit water conditions (F3 to F7). All five control varieties underwent cultivation under both conditions. These findings underscore the necessity for tailored breeding programs attuned to specific environmental exigencies, recognizing that individual traits manifest divergent responses to varying conditions. It is evident that certain traits exhibit marked disparities under well-watered conditions, while others evince heightened differentiation under water deficit conditions. Significantly, our analysis reveals a pronounced interaction between irrigation regimes and selection traits, which serves to underscore the nuanced interplay between genotype and environmental stress.

## Introduction

Groundwater resources decreasing gradually because of global climate change, escalating energy costs and increased demand for water for both industrial and human consumption has led to a reduction in the amount of water available for agricultural production. At the same time, one of the most noticeable effects of global warming, particularly in recent years, the negative impact of drought on crop productivity. Although cotton (*Gossypium hirsutum* L.) shows a certain degree of drought tolerance compared to other crops, the decline in cotton production can be high, reaching up to 70–80%, depending on the duration and timing of the drought (Ray, Fares & Risch, 2018).

Other studies have emphasized the negative effects of water stress on both cotton productivity and fiber quality. The decline in crop production due to water stress in cotton is dependent on the specific growing season and the intensity of the drought. The most sensitive period of cotton plants to water stress is the beginning of the first white flowers. Additionally, drought has the most significant effect on yield during the peak flowering period (Bozorov et al., 2018; Krieg, 1997). The main factor contributing to reduced cotton production during drought is the decrease in boll number per plant (Pettigrew, 2004; Basal et al., 2009). Both production and fiber quality are adversely affected by drought. Reports suggest that drought during the elongation phase of cotton fibers negatively impacts length, strength, and maturity (Johnson et al., 2002; Ritchie et al., 2004; McWilliams, 2003; Mert, 2005). According to McWilliams (2003), drought during the later stages of blooming hinders boll growth, resulting in a higher percentage of premature fibers with reduced strength.

In order to improve drought tolerance, it is important to identify the selection criteria that contribute to drought tolerance and to use the genetic variation found within populations to create cultivars that can withstand drought conditions. Turner (1986) recommended several breeding strategies, including the selection of cultivars with high productivity even under stressful conditions. These strategies include (1) Selection for high yield under stress conditions: However, this approach has a significant drawback. Crop yields can fluctuate widely from year to year, resulting in slow genetic progress due to small changes in both yield and other traits (Ludlow & Muchow, 1990). (2) *Integrated selection through physiological and morphological mechanisms:* This methodology utilizes traditional breeding procedures and is based on qualities known to provide tolerance to water stress (Foulkes, Scott & Sylvester Bradely, 2001). (3) *Character-based selection for drought resistance:* This method focuses on identifying a specific characteristic that contributes to drought resistance. It involves choosing plants with high production under drought-induced stress conditions based on a particular trait (Merah, 2001). Each of these strategies contributes to the continuous attempt to cultivate crop varieties that are resilient to drought, offering substantial options to mitigate the effects of water scarcity on crop production.

Drought tolerance in cultivars can be achieved through different breeding methods, each with its own approach and focus. The concept of selection under optimal conditions suggests that plant lines with high yields in favorable environments will also have good yields in difficult environments (Weber et al., 2012). On the other hand, direct selection in drought conditions involves two methodologies: indirect selection, which comprehensively evaluates physiological, morphological, and yield components, and direct selection, which primarily focuses on yield (Ziyomo & Bernardo, 2013). Indirect selection for developing high-yielding, drought-tolerant varieties should be conducted under drought-stress conditions (Ebrahimiyan, Majidi & Mirlohi, 2012). Additionally, indirect selection under low and optimal conditions was found to be more efficient than direct selection under random abiotic stress or indirect selection under managed drought, especially for early maturing genotypes, but direct selection was most efficient for predicting performance under low (Weber et al., 2012). This suggests that the choice of breeding method depends on the specific environmental conditions and the traits being targeted.

However, Piepho (2000) indicates that the direct selection method under water stress hindered genetic progress due to genotype-environment interactions, epistatic gene effects and low heritability. On the other hand, Rizza et al. (2004) and Ober et al (2005)’s studies on barley and sugar beet indicated that mean yields of various varieties under well-watered conditions and different locations enhanced the success of selection. Notably, those varieties demonstrating high yield capacity under favorable circumstances also exhibited promising yield performance in drought conditions. Voltas, Lopez-Corcoles & Borras (2005) recommended considering yield performance under different environmental conditions as the primary important for developing drought-tolerant varieties. Because of challenges associated with utilizing physiological traits as dependable indicators for yield under drought stress conditions. According to Ceccarelli (1996), lines that were cultivated in ideal conditions do not perform well when exposed to stressful environmental conditions, whereas varieties that were chosen for their stress resistance had lower yields when grown in ideal conditions (Rosielle & Hamblin, 1981). Other studies consistently emphasize that selection would give better results if conducted at initial generation in optimal conditions (Quisenberry et al., 1980). Conversely, Shakoor et al. (2010) proposed that the process of selecting drought-tolerant plants should be conducted in conditions of water stress.

The different opinions of abiotic stress tolerance breeding programs that have been going on for years are mentioned above. However, the exact solutions have not been determined. There are advantages and disadvantages for each approach. In this study, we have examined all the existing approaches and compared both optimum and stress conditions in terms of genotypes and selection traits with different statistical analyses. The aim here is to clarify the current confusion.

There are one primary and three secondary goals of this study: main objective (1) evaluate whether the selection process should be carried out under well-watered (optimal) conditions or water deficit (stressful) conditions to increase the overall success of breeding programs that target drought-tolerant cultivars; (2) analyze and compare the performances of different cotton lines which developed both under well-watered and deficit water conditions; (3) determine the most effective selection criteria for use under deficit water conditions; (4) identify the best drought-tolerant lines.

This study comprehensively analyzed the advantages and disadvantages of each approach. The findings have both theoretical and practical importance, as they can be immediately can applied to real-world scenarios. These findings provide crucial insights that can optimize drought breeding programs and findings are expected to make a significant contribution to the academic field by advancing our understanding of drought breeding programs and the development of drought-tolerant cotton varieties.

## Materials and Methods

The study began in 2008 by using line tester method with five cotton varieties which are most cultivated and high yielding in Turkey (Carmen, STN 453, Şahin 2000, GSN 12, BA 119) and seven varieties (Tamcot 22, SJ U86, DPL 90, NIAB 999, NIAB 111, Eva, AZ 31) which are cultivated in different countries. The characteristics of these variations are presented at the Table S1.

**Figure 1:**
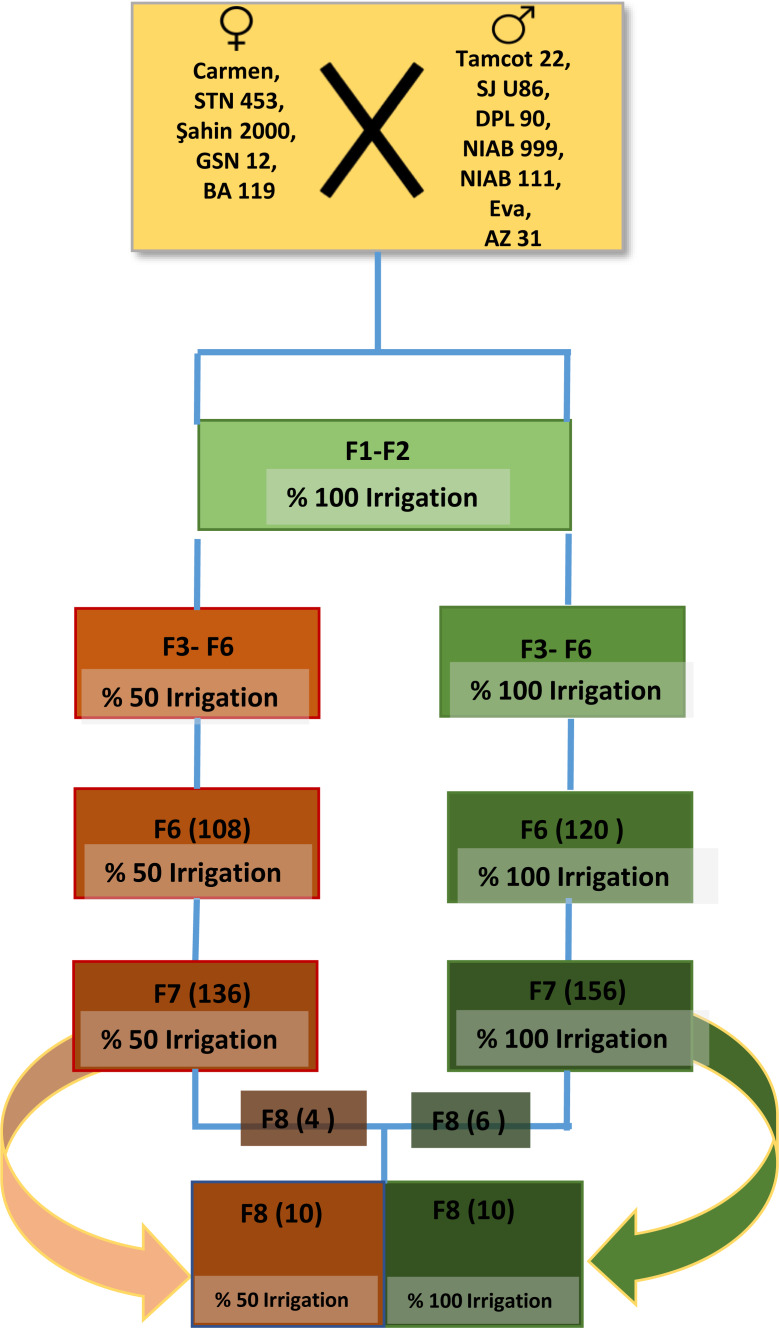
The breeding process for drought-resistant plants is demonstrated in this flowchart.

The crosses were done using the line tester method at the Faculty of Agriculture, Adnan Menderes University, Turkey. The F1 and F2 generations were grown in 2009 and 2010, respectively. The F3 generation was formed from the seeds obtained from the bolls taken from the hybrid combinations (35 hybrid combinations), forming the F2 generation by the single boll method. The seeds obtained from the bolls taken by the single boll method in the F2 generation were divided into two parts. F3 generation was formed in 2012 in order to grow half of these seeds under well-watered (100% irrigation) and the other half under deficit (50% irrigation) conditions. In the F3 generation, hybrid combinations and parents were grown with five control varieties with four blocks under full and deficit irrigation conditions. Single plant selection was started in the F3 generation. In 2012, single plants selected from the F3 generation which grown under full and limited irrigation conditions were transferred to the F4 generation. F4 In 2013, F5 in 2014, and F6 in 2015 generations were conducted. Single plant selections from F3 to F6 generation were carried out separately under well-watered and deficit water conditions in an augmented experimental design with four blocks and 12 m length. Single plant selection from F3 generation to F6 generation under deficit and full irrigation conditions was carried out in two stages as yield and fiber quality parameters. 88 full irrigation hybrid progeny lines and 100 limited irrigation hybrid progeny lines were transferred to the F6 generation in 2015. Overall, the F6 and F7 generations of the study were carried out under well-watered (100%) and deficit water (50%) conditions with four blocks in Augmented experimental design, single plant progeny rows with five control varieties (K1:Karizma, K2:Gloria, K3:Carla, K4:Candia and K5:Claudia) in one row and 12 m in length (Fig. 1).

### Experimental design

In this article, we evaluate these ten homozygous lines in a randomized split-block design with five control varieties (Candia, Carla, Claudia, Gloria, Charisma) with four replicates in both well-irrigated and deficit water conditions (F8 generation), with main plots being irrigation sub-plot lines. In this article, we evaluate these ten homozygous lines in a randomized split-block design with five control varieties (Candia, Carla, Claudia, Gloria, Charisma) with four replicates in both well-irrigated and deficit water conditions (F8 generation), with main plots being irrigation sub-plot lines. Six of the ten homozygous lines (LTNS-73, LTNS-24, LTNS-117, LTNS-142, LTNS-34, and LTNS-59) were only grown under well-watered conditions (F3 to F7), while the remaining four lines (LTDS-116, LTDS-128, LTDS-130, LTDS-34) were only grown under deficit water conditions (F3 to F7). All five control varieties (Candia, Carla, Claudia, Gloria, and Karizma) were grown under both well-watered and deficit water conditions (Fig. 1).

Observations were recorded on five randomly selected each line were used for observing the following traits Observations, boll weight (g), ginning percentage, and seed cotton yield (Kg/da). Ginning percentage was calculated by using formula suggested by Ghule et al. (2013)

Ginning percentage (%) = (Weight of lint/Weight of seed cotton) x 100.

We collected three samples from each line for fiber quality analysis after the lint cleaner. I measured the fiber quality traits with HVI instruments (fiber length, elongation, micronaire, strength).

### Irrigation method

The experiment included two irrigation regimes, well-watered (100% field capacity) and deficit irrigation (50% field capacity). Well-watered (control treatment) was designed to provide 100% replenishment of soil water depletion. A surface drip irrigation system was used for irrigation. A 16 mm diameter polyethylene pipe with in-line pressure compensating drippers at 0.33 m intervals was placed on one side of each cotton row. The different irrigation treatments were started on 10 June and continued according to regional practice until late August when 10% of bolls on a plant were fully open. The average amount of water applied was 380 mm for 50% (deficit water) and 760 mm for 100% (well-watered). Soil water levels were monitored by gravimetric method in the plots.

### Statistical analysis

The data was analyzed using the ANOVA model (Analysis of Variance) with a split-plot experimental design. The evaluation was performed to determine the productivity of several cotton genotypes in terms of seed cotton production, considering both well-watered (optimal) and deficit-water (stress) conditions. In addition, a pairwise comparison analysis was performed to evaluate the influence of well-watered and deficit-water conditions on the seed cotton yield. Analyses revealed mean yield differences, which were quantified using a Student’s *t*-test. Cotton lines were subjected to a two-way heatmap cluster analysis on features associated with fiber quality and yield traits under well-water and deficit-water conditions. The JMP^®^ software version <16.0> developed by SAS Institute Inc, Cary, NC, from 1989 to 2023, was used. The Analysis of Means (ANOM) chart presents decision thresholds for boll weight and fiber strength in both drought and well-water conditions with a significance level of alpha at 0.05 The SigmaXL analysis software was employed to conduct a graphical test for the simultaneous comparison of the mean performance of cotton genotypes under well-water and drought stress conditions. Pearson’s correlation coefficient computed with R-package program (R-4.3.3 for Windows)with “metan” (Anderson, 2003).

## Results

ANOVA results in Table 1 showed significant effects of irrigation on LP (lint percentage), BW (boll weight), YD (seed cotton yield) and FF (fiber fineness). Additionally, genotypes exhibited variations in LP, BW, FF, FS (fiber strength) and FE (fiber elongation) features. The interaction between genotype and irrigation was determined to have a substantial impact on the LP, BW, FF, FL (fiber length), FS, and FE characteristics (Table 1).

### Decision chart/analysis of mean methods

**Figure 2:**
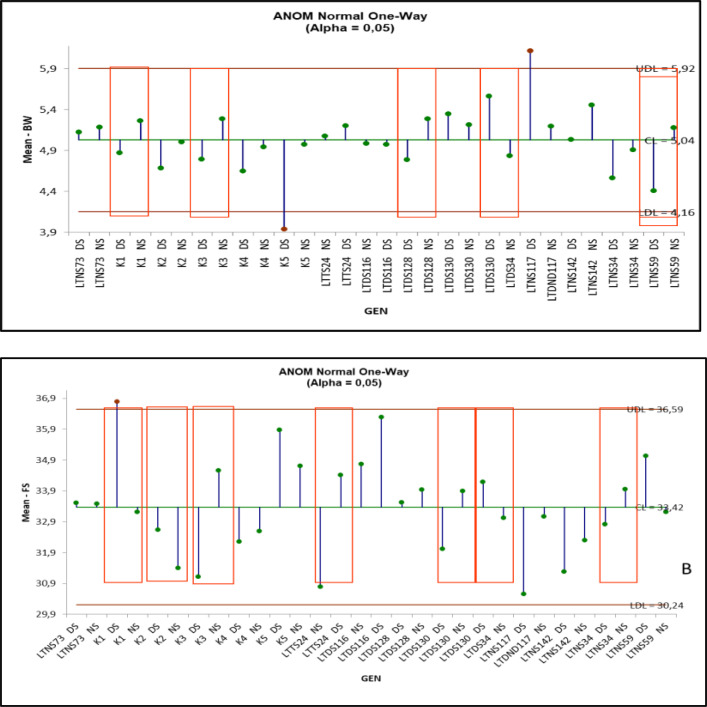
ANOM-decision chart with decision limits for boll weight (A) and fiber strenght (B) across drought stress and well water (alpha > 0.05%). It provides a graphical test for simultaneously comparing the mean performance of these cotton genotypes across drought stress and well water. Red-colored heads represent significant deviation from the mean, either above upper decision level (UDL) or below lower decision level (LDL).

The findings from the initial examination of study conducted under well-watered and deficit water conditions are displayed in Table 1. According to the results, there was an interaction between genotype and irrigation for BW (boll weight) and FS (fiber strength). The genotypic behavior for selection based on analysis of means (ANOM) was represented by plotting the results of mean comparisons by using ANOM. According to the data presented in Figs. 2A and 2B. The genotypes within the red square in the ANOM analysis exhibited dynamic characteristics when subjected to both well-watered and deficit irrigation conditions. For instance, in case of the BW trait, the LTDS- 34 genotype exhibited values that were higher than the average under well-watered irrigation conditions and lower than the average under deficit irrigation conditions (Fig. 2A). While examining the fiber strength trait, we observed that seven genotypes exhibited values that varied from the standard. On the other hand, in terms of the FS characteristic, genotype K1, which is one of the control varieties, has the most significant response compared to the two-irrigation condition. Upon evaluating the cultivars for their tolerance to deficit irrigation, it was observed that LTDS- 117 exhibited the highest tolerance among the genotypes for BW, while LTDS- 116 showed a better tolerance for FS (Fig. 2B).

**Table 1:** Analysis of variance of the performance of lines grown under full and deficit irrigation conditions.

**Source**	**DF**	**LP**	**BW**	**YD**	**FF**	**FL**	**FU**	**FS**	**FE**
**Rep**	**3**	0.69	0.26	12422.60	0.15	2.17	2.41	0.86	0.11
**Irrigation**	**1**	32.76^**^	1.18	314651^**^	1.75^**^	59.04^**^	0.21	6.26	0.36
**Genotype**	**14**	5.26^**^	0.64^**^	4552.77	0.11^**^	2.42	2.64	11.71^**^	1.17^**^
G*I	**14**	9.25^**^	0.52^**^	7752.76	0.12^**^	3.97^**^	2.76	8.13^**^	2.25^**^

**Notes.**

LP, Lint percentage; BW, Boll weight; YD, Cotton seed yield; FF, Fiber fineness; FS, Fiber strength; FL, Fiber length; FU, Fiber uniformity; FS, Fiber strength; FE, Fiber elongation.

** *P*-value is less than 0.01 (*p* < 0.01).

* *P*-value is generally less than 0.05 (*p* < 0.05).

### Percentage changes in traits under full and deficit irrigation in the same year and field conditions for breeding program varieties

Table 2 shows the mean values and percentage differences for ten cotton genotypes, grown under well-watered and deficit water conditions. According to the results, the genotype with the highest lint percentage was found LTDS- 34 with 41.38% in well-watered conditions, while LTNS- 142 with 43.98% in deficit water conditions. Considering the changes in ginning yield under deficit water, LTNS-142 showed the highest positive change with 13.48%, followed by LTDS-128 with −6.11%. For boll weight feature, LTNS- 142 (5.47 g) under well-watered conditions and LTSS 117 (6.13 g) under deficit water conditions showed the highest value. The highest differences were LTNS- 117 (+) 17.66%; LTDS- 34 (+) 15.05%; LTNS- 59 (-)14.84%, respectively. For seed cotton yield, ıt is important to take note that the direction of change that has been identified for all lines, except for LTDS- 128, has been observed to be in a negative direction. The most severe declines in production were seen in lines that were established under conditions of well-watered. Specifically, LTNS- 73 (-35.5%) and LTNS- 59 (-31.03%) had the biggest yield losses. In terms of the fiber strenght, shifting from conditions of well-watered to conditions of deficit irrigation led to a reduction in the fineness of the fibers. The greatest response to water stress was seen in LTNS- 59, which had a rise in coarseness of 14.70% after being exposed to it. With well-watered, fiber length was shown to be greater, but it decreased under low irrigation conditions. The LTNS- 142 line had the most significant negative response, with a decrease of -11.45%. On the contrary, the LTKD 34 line showed the biggest positive change, with an increase of 4.54%. The fiber uniformity (FU) measurement exhibited the highest level of stability in terms of fiber quality, regardless of whether the irrigation circumstances were full or deficit . The LTNS- 74 line showed the most significant decrease in fiber strength (FS) measurement, with a negative difference of 10.48% when comparing well-watered to reduced irrigation. An increase in LTNS- 34 and LTDS- 34 lines was seen. The fiber elongation (FE) a quality trait is significantly affected by drought applications. However, this influence has been observed in an inconsistent pattern. For instance, although the LTDS- 116 line was affected by 25.02%, the LTNS- 142 line was affected by a positive 21.96%.

**Table 2:** Percentage changes of the traits under well-watered (bold) and deficit irrigation in the same year and under the same field conditions of the varieties whose breeding programs were conducted under well-watered and deficit irrigation (italic).

**Gen**	**LP**			**BW**			**YD**			**FF**		
	**NS**	**DS**	**Differ.**	**NS**	**DS**	**Differ.**	**NS**	**DS**	**Differ.**	**NS**	**DS**	**Differ.**
			**(%)**			**(%)**			**(%)**			**(%)**
**LTDS- 116**	40.36	40.20	**−0**.**38**	4.99	5.00	**0**.**20**	454.76	454.15	**−0**.**13**	4.72	5.08	**−7**.**72**
**LTDS- 128**	40.13	37.68	**−6**.**11**	5.30	4.80	**−9**.**43**	445.77	455.45	**2**.**17**	5.02	5.14	**−2**.**47**
**LTDS- 130**	39.82	38.75	**−2**.**69**	5.23	5.36	**2**.**49**	439.71	394.92	**−10**.**19**	4.78	4.82	**−0**.**83**
**LTDS- 34**	41.38	39.51	**−4**.**52**	4.85	5.58	**15**.**05**	492.85	369.37	**−25**.**06**	5.02	4.95	**1**.**29**
***LTNS- 34***	40.08	42.15	**5**.**16**	4.92	4.58	**−6**.**91**	505.35	425.26	**−15**.**85**	4.80	5.08	**−5**.**85**
***LTNS- 59***	39.85	41.39	**3**.**85**	5.19	4.42	**−14**.**84**	543.40	374.77	**−31**.**03**	4.74	5.43	**−14**.**60**
***LTNS- 73***	40.06	42.79	**6**.**81**	5.20	5.14	**−1**.**15**	523.16	337.45	**−35**.**50**	5.11	5.20	**−1**.**85**
***LTNS- 74***	39.51	41.66	**5**.**44**	5.22	5.09	**−2**.**49**	480.83	393.49	**−18**.**16**	4.79	5.21	**−8**.**72**
***LTNS- 117***	40.90	40.08	**−2**.**01**	5.21	6.13	**17**.**66**	513.71	377.94	**−26**.**43**	4.90	4.80	**1**.**98**
***LTNS- 142***	38.75	43.98	**13**.**48**	5.47	5.05	**−7**.**68**	497.63	449.71	**−9**.**63**	4.86	5.30	**−9**.**08**
**Gen**	**FL**			**FU**			**FS**			**FE**		
	**NS**	**DS**	**Differ.**	**NS**	**DS**	**Differ.**	**NS**	**DS**	**Differ.**	**NS**	**DS**	**Differ.**
			**(%)**			**(%)**			**(%)**			**(%)**
**LTDS- 116**	31.05	29.93	**−3**.**58**	86.08	86.20	**0**.**15**	36.33	34.81	**−4**.**17**	6.39	4.79	**−25**.**02**
**LTDS- 128**	30.47	30.45	**−0**.**05**	85.42	86.44	**1**.**19**	33.98	33.56	**−1**.**22**	6.00	6.06	**1**.**00**
**LTDS- 130**	30.17	29.72	**−1**.**49**	85.29	84.93	**−0**.**41**	33.93	32.05	**−5**.**53**	5.98	4.89	**−18**.**25**
**LTDS- 34**	29.39	30.72	**4**.**54**	84.14	87.09	**3**.**51**	33.06	34.24	**3**.**57**	6.22	5.59	**−10**.**05**
***LTNS- 34***	31.20	30.78	**−1**.**36**	86.35	85.98	**−0**.**42**	34.00	32.86	**−3**.**34**	5.95	5.07	**−14**.**76**
***LTNS- 59***	31.28	29.55	**−5**.**55**	85.16	85.82	**0**.**78**	33.26	35.07	**5**.**45**	5.12	5.53	**8**.**16**
***LTNS- 73***	30.17	29.89	**−0**.**90**	85.06	85.07	**0**.**01**	33.52	33.56	**0**.**10**	5.79	5.72	**−1**.**27**
***LTNS- 74***	31.80	29.61	**−6**.**89**	86.48	86.13	**−0**.**41**	34.45	30.84	**−10**.**48**	5.19	5.94	**14**.**43**
***LTNS- 117***	31.05	29.94	**−3**.**57**	85.90	83.63	**−2**.**64**	33.11	30.60	**−7**.**57**	5.64	5.78	**2**.**55**
***LTNS- 142***	30.99	27.44	**−11**.**45**	85.50	84.44	**−1**.**24**	32.34	31.32	**−3**.**17**	5.54	6.76	**21**.**96**

**Notes.**

LP, Lint percentage; BW, Boll weight; YD, Cotton seed yield FF; Fiber fineness; FS, Fiber strength; FL, Fiber length; FU, Fiber uniformity; FS, Fiber strength; FE, Fiber elongation. *LTNS lines were bred at 100% irrigation and LTDS lines were bred at 50% irrigation.*

### Cluster analysis

Estimation of dissimilarity among the experimental genotypes based on agronomic and fiber-related traits under optimal and water stress conditions was performed by using agglomerative hierarchical clustering (AHC) analysis. The AHC analysis employed a “bottom-up” technique to create a cluster tree. Initially, each observation was treated as its own cluster and then paired successively to form a hierarchy until distinct clusters were formed. The Euclidean distance method was used to calculate the distances between pairs of genotypes. Ward’s method was used to create a dendrogram that represented the complete clustering of all the genotypes. A two-way clustering technique was utilized to construct a two-way cluster diagram through AHC.

**Figure 3:**
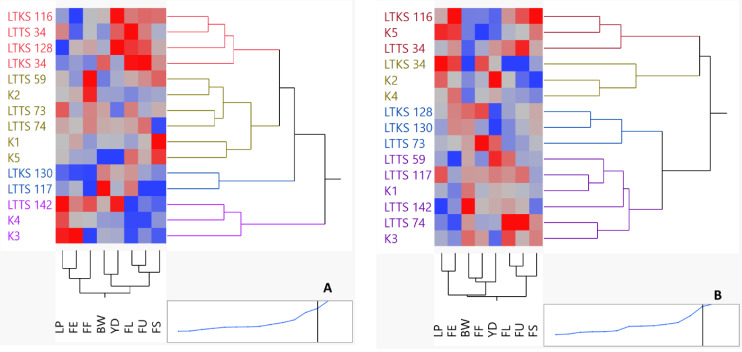
Hierarchical clustering of cotton genotypes for agronomic and fiber-related traits under drought stress (DS) (A) and well water(NS) (B) conditions. LTNS lines were bred at 100% and LTDS lines were bred at 50%.

The AHC analysis categorized all 15 genotypes (five control and 10 variation) into four groups, both under optimal (well-watered) and deficit water (drought) conditions, as shown in Fig. 3A. Particularly, during deficit water period, group-1, group-2, group-3 and group-4 included two, six, four, and three genotypes, respectively. In the case of well watered condition, the genotypes were categorized into four groups as well: group-1, group-2, group-3, and group-4. These groups consisted of three, seven, three, and two genotypes, respectively (Fig. 3B). Each cluster was assigned a different color for better visualization.

Based on the color gradient generated by clustering, it was determined that specific genotypes had beneficial characteristics that linked to their performance. Specifically, under deficit water conditions, genotypes such as LTNS-142, LTNS-34, LTDS-116, LTDS-128 and LTDS-34 showed greater performance in terms of agronomic and fiber quality features. Similarly, under well watered conditions, genotypes LTDS-116, LTDS-34, and LTNS-74 showed favorable characteristics.

The heat map resulting from the cluster analysis of the eight traits shown in Figs. 3A and 3B on the horizontal axis grouped the traits into three agronomic and five fiber quality groups. A visual inspection of the heat map shows that the pattern of values for YD (seed cotton yield) and BW (boll weight) were in the same group under both well-watered and deficit water conditions, whereas FU (fiber elongation) and F (fiber length) were found to be in the same group under both well-watered and deficit water conditions. For the well watered conditions, the FS (fiber strength) and FU (fiber uniformite) trait was sorted into different colors ranging from dark blue to deep red. For this reason, the use of FS as traits to discriminate genotypes for full irrigation will provide important convenience. During a period of water deficit the traits BW and FS were found to be separating traits. It is worth mentioning that LTNS-117 displayed a yellow to far yellow color for BW, showing high values. On the other hand, K5 showed a red to far red color, showing low values, for the same characteristics. This pattern of colors reveals an important distinction between these two lines in most of the characteristics (Fig. 3A).

### Cotton seed yield comparison of homozygous lines under full and deficit irrigation: pairwise comparison analysis

**Figure 4:**
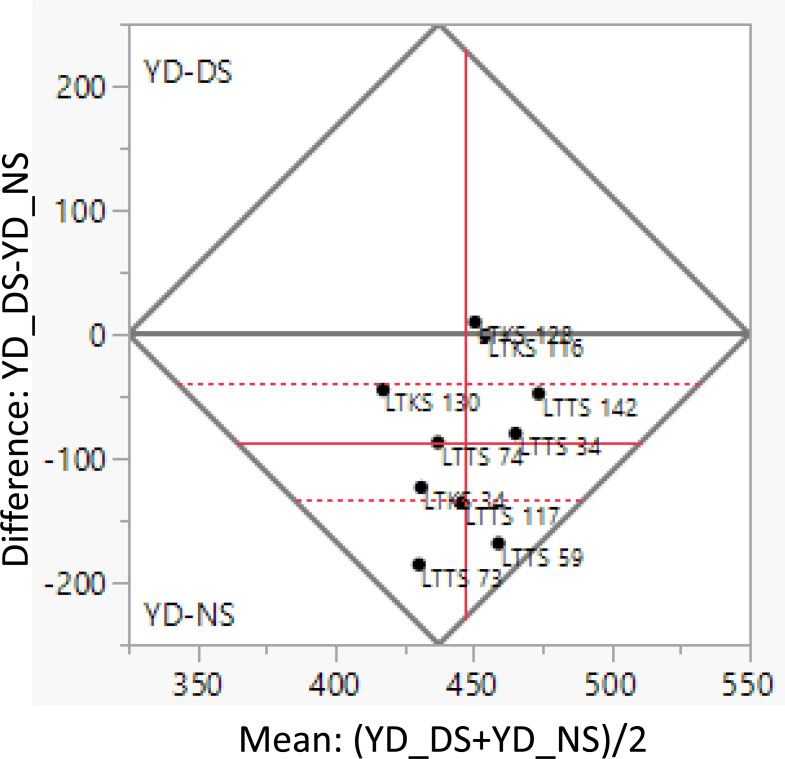
Means comparison and histograms of homozygous lines grown under well-watered and deficit irrigation conditions using pairwise comparison analyses and *t*-test method with a probability level of 0.05.

Comparing normal irrigation and deficit irrigation in terms of their impact on yield characteristics, it was found that the average difference between the genotypes under deficit irrigation and well-watered was 86.46 kg/da (Table 3) The observed discrepancy was determined to have statistical significance. Based on the findings, the lines LTDS-130, LTDS-34, LTNS-74, 142, and 34 exhibited normal distribution, while LTDS-116 and LTDS-128 (chosen during the breeding program under deficit irrigation) remained unaffected by deficit irrigation in terms of yield and even exceeded the upper 95% limit. In contrast, the LTNS-59 and LTNS-73 lines, which were chosen during the breeding program under optimal irrigation conditions, were observed to fall below the 95% lower threshold, unlike the other lines. Consequently, these lines were identified as the ones most impacted by deficit irrigation conditions. Overall although showing some yield reduction under deficit irrigation, lines selected under restricted irrigation were less impacted compared to lines selected under well-watered. This trend suggests that the former lines are more suitable for making a healthier selection. Line LTNS-142 exhibits the highest average seed cotton yield, while experiencing yield reduction when subjected to deficit conditions (Fig. 4).

**Table 3:** Results of pairwise comparison analysis of yield variation under full and deficit irrigation conditions.

**YD-DS**	403.251	**t-Ratio**	−4.08727
**YD-NS**	489.719	**DF**	9
**Mean difference**	−86.468	**Prob < —t—**	0.0027^**^
**Std error**	21.1555	**Prob > t**	0.9986
**Upper 95%**	−38.611	**Prob < t**	0.0014^**^
**Lower 95%**	−134.33		
**Correlation**	−0.5773		

**Notes.**

** *P*-value is less than 0.01 (*p* < 0.01).

* *P*-value is generally less than 0.05 (*p* < 0.05).

Table 4 displays the average differences in yield values between the lines cultivated under complete and restricted irrigation. The differences were analyzed using *t*-test. The LTDS- 116 and LTDS- 128 lines exhibited no statistically significant variation in the yield trait, regardless of whether they were subjected to well-watered or reduced irrigation circumstances. Furthermore, these lines showed consistent and reliable performance. Tolerant, in the context of organisms or plants, denotes the capacity to withstand or endure specific conditions or influences. This refers to the ability to tolerate or mitigate the adverse impacts of different stressors. According to this criterion, the LTDS- 116 and LTDS- 128 lines can be classified as “drought stress tolerant” compared to other lines because they continuously maintain their yield performance even under deficit irrigation conditions It’s important to note that these two lines were specifically bred under stress conditions (with 50% irrigation), which highlights the study’s premise.

**Table 4:** Method of t-test analysis of seed cotton yield (YD) variation under full and deficit irrigation conditions. LTNS lines were bred at 100% and LTDS lines were bred at 50%.

**Genotype**	**Mean difference** **YD**	**Genotype**	**Mean difference** **YD**
**LTDS- 34**	−123.5^**^	**LTNS- 34**	−80.09^**^
**LTDS- 116**	−0.61	**LTNS- 59**	−168.6^**^
**LTDS- 128**	9.6723	**LTNS- 73**	−185.7^**^
**LTDS- 130**	−44.79^**^	**LTNS- 74**	−87.34^**^
**LTNS- 142**	−47.92^**^	**LTNS- 117**	−135.8^**^
**Critical value (t test)**		**0.05**	−7.49197
		**0.01**	−11.5302

**Notes.**

** *P*-value is less than 0.01 (*p* < 0.01).

* *P*-value is generally less than 0.05 (*p* < 0.05).

The ANOVA analysis comparing line averages under different irrigation conditions (well-watered or deficit irrigation) revealed significant environmental effects on lint percentage (LP), boll weight (BW), and fiber fineness (FF) traits. Specifically, the LP trait demonstrated the highest average under well-watered conditions compared to BW and FF. This indicates that LP is particularly responsive to irrigation levels, showcasing its potential as a trait influenced by environmental conditions, particularly under optimal watering conditions (well-watered). These findings underscore the importance of considering irrigation management in cotton breeding programs to optimize trait expression and ultimately enhance crop performance.

A comparison of line averages, breeding with well-watered or deficit irrigation, and LSD is presented in detail in Tables 5 and 6.

**Table 5:** Comparison of line averages, breeding with well-watered or deficit irrigation.

**Source**	**DF**	**Mean Square**							
		**LP**	**BW**	**YD**	**FF**	**FL**	**FU**	**FS**	**FE**
**ENV.**	**1**	70.07^**^	1.14^**^	10211.60	1.72^**^	0.50	1.37	2.74	0.44
**Block**	**23**	3.03	0.50	7096.37	0.13	1.89	4.05	5.81	0.55
**Error**	**15**	1.52	0.07	5633.15	0.03	2.50	2.04	7.54	0.31

**Notes.**

LP, Lint percentage; BW, Boll weight; YD, Cotton seed yield; FF, Fiber fineness; FS, Fiber strength; FL, Fiber length; FU, Fiber uniformity; FS, Fiber strength; FE, Fiber elongation.

** *P*-value is less than 0.01 (*p* < 0.01).

* *P*-value is generally less than 0.05 (*p* < 0.05).

**Table 6:** LSD for comparison of line averages, breeding with well-watered or deficit irrigation.

**ENV.**	**LP**	**BW**	**YD**	**FF**	**FL**	**FU**	**FS**	**FE**
**LTNS**	42.01 A	5.07 B	393.10	5.17 B	29.54	85.18	32.37	5.80
**LTDS**	39.05 B	5.45 A	428.83	4.94 A	29.79	85.59	32.96	5.57
**LSD**_**(0.05)**_	**0.9291**	**2.0429**		**0.1359**				

**Notes.**

LP, Lint percentage; BW, Boll weight; YD, Cotton seed yield; FF, Fiber fineness; FS, Fiber strength; FL, Fiber length; FU, Fiber uniformity; FS, Fiber strength; FE, Fiber elongation; ENV, Well-watered or deficit condition; LTNS, Line Tester Normal Condition; LTDS, Line Tester Drought Condition.

LTNS lines were bred at 100% and LTDS lines were bred at 50%.

### Correlation analysis

**Figure 5:**
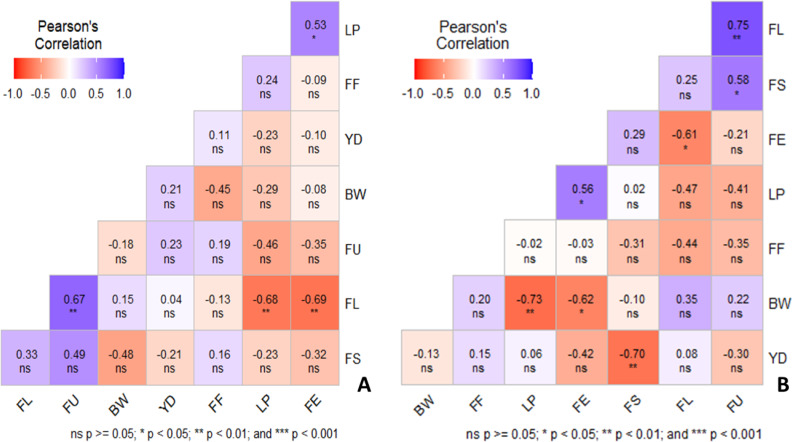
Correlation coefficients between yield and yield components of cotton under water deficit (A) and well-watered (B) conditions.

Correlation coefficients between yield and yield components of cotton under water deficit (left) and well-watered (right) conditions are presented in Fig. 5. This analysis was carried out to measure whether the correlations are important to use for selection traits changed under full and deficit irrigation conditions.

As shown in Fig. 5A, fiber length (FL) was positively correlated with fiber uniformity (FU), while fiber length (FL) was negatively correlated with micronaire (LP), and both FL and elongation (FE) were positively correlated with yield (YD) under deficit irrigation conditions. On the other hand, FU was positively and significantly correlated with FS and FL; FL and FU negatively and significantly; BW and LP was negatively and significantly; LP and FE positively and significantly correlated with well-watered condition. However, YD negatively and significantly correlated with FS under well-watered condition (Fig. 5B) This trait, which is important for us, showed statistically different behavior under different irrigation conditions. FS was found to be the most important trait affecting the fiber traits under full and deficit irrigation conditions and did not change against stress conditions. The results of correlation coefficient analysis and cluster analysis support each other.

### Principal component analysis

Principal component analysis (PCA) stands as a robust statistical methodology employed for the examination and simplification of intricate and expansive datasets. Within the scope of this investigation, PCA was applied to scrutinize the variation patterns inherent in cotton genotypes, with a specific focus on evaluating their genetic diversity concerning the studied traits

The PCA analysis revealed that eight principal components (PCs) explained the total variation in both well-watered and deficit irrigation conditions.

**Figure 6:**
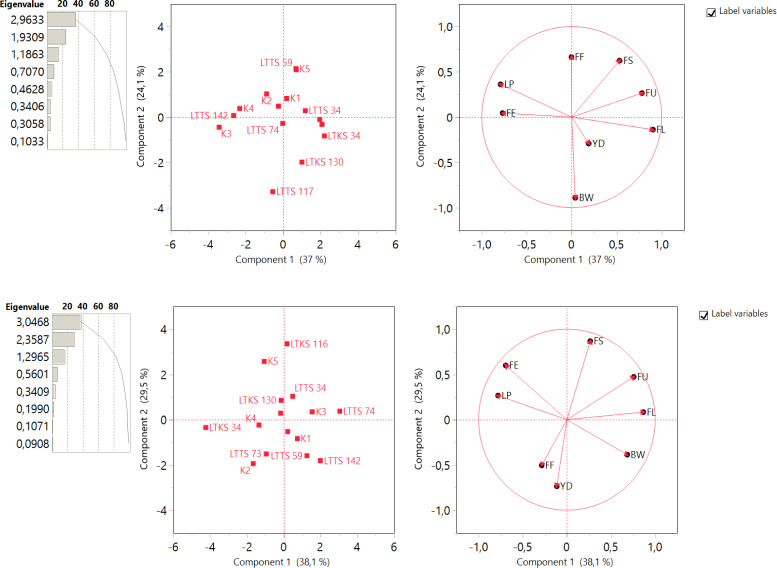
Summary of bar chart displaying eigenvalue and variation percentage contribution by all principal components (PCs), a biplot between PC1 and PC2 displaying the distribution of 10 lines and five check cotton genotypes under deficit irrigation (A) and well-watered irrigation (B) condition for F8 generation.

For the genotypes grown under 50% deficit conditions, the first two principal components (PCs) were significant in explaining the variation among the genotypes. The cumulative contribution of the first three PCs accounted for 75.928% of the total variability in selection parameters, indicating their strong association with these traits under deficit irrigation conditions (Fig. 6A). Under well-watered conditions (100%), the first three principal components (PCs) were found to have eigenvalues greater than 1, indicating their relevance in explaining the observed variation .The cumulative contribution of the first three PCs accounted for 83.775% of the total variance in yield and fiber quality traits under well watereded conditions (Fig. 6B).

The summary biplots of studied traits, along with their magnitudes of variation, were analyzed to assess trait correlations and genetic diversity in cotton genotypes. The summary biplot displayed in Fig. 6 revealed that all genotypes under stress condition were distributed within the correlation eclipse formed by the first two Principal Components (PCs) (Fig. 6A, left). The second summary biplot in Fig. 6A right, between PC-1 and PC-2, explained 61.1% of the total variation. Trait correlations were observed: FS and FU,positive correlations with each other; BW and YD positive correlations while they exhibited negative correlations with FF and LP. The vector lengths originating from the center depicted the strength of correlations among traits, validating the mentioned correlations under drought conditions In terms of variability, the traits BW and FL displayed long vectors, indicating higher variation, while YD exhibited the least variability.

The summary biplot displayed in Fig. 6 revealed that all genotypes under well watereded condition were distributed within the correlation eclipse formed by the first two Principal Components (PCs) (Fig. 6B, left). The second summary biplot in Fig. 6B right, between PC-1 and PC-2, explained 67.6% of the total variation. Trait correlations were observed: FS, FL and FU positive correlations with each other; FF and YD positive correlations while they exhibited negative correlations with FS and FU. The vector lengths originating from the center depicted the strength of correlations among traits, validating the mentioned correlations under well watereded conditions In terms of variability, the traits FS, FU and FL displayed long vectors, indicating higher variation, while FF exhibited the least variability

## Discussion

Our study found that genotypes cultivated under water deficit (stress) conditions exhibited higher lint percentages compared to the genotypes grown under well-watered (optimal) conditions. This difference can be related to seed weight because as water doses decrease in cotton production, seed weight also decreases so lint percentages escalate as indicated by other researchers (Ertek & Kanber, 1994; Pettigrew, 2004; Balckhom et al., 2006; Basal et al., 2009). Furthermore, the results of this study support the hypothesis that the ginning yield is increasing under deficit water conditions, which is consistent with other studies conducted by Pettigrew (2004), Mert (2005), Rai (2011), and Cave (2013).

According to Marani &Amirav (1971), Marani (1973), Mert (2005) and Başal et al. (2009), boll weight decreases when cotton is exposed to water deficit conditions. However, Pettigrew (2004) found that water stress did not have a significant effect on the weight of an individual boll. In contrast, Önder et al. (2009) found that increased water availability had a positive impact on boll weight.

Hussein, Janat & Yakoub (2011) and Kang et al. (2012) found that the boll weight had a different reaction to different irrigation regimes. The mean yield values of cultivars grown under well-watered irrigation conditions were significantly greater than those grown under deficit water conditions. Our study’s findings correlate with previous researchers who also observed a decrease in crop productivity due to water stress (Tekinel & Kanber, 1978; Krieg, 1997; Krieg, 2000; Ertek & Kanber, 1994; McWilliams, 2003; Pettigrew, 2004; Mert, 2005; Balckhom et al., 2006; Rai, 2011).

Under deficit irrigation regimes, Mert (2005) reported that water stress positively affected fiber fineness in his study, while Basal et al. (2009) and Price (2009) reported that water stress negatively affected fiber fineness in their related studies, similar to our study. According to most of the previous studies, fiber length shortens with increasing water stress levels (Marani, 1973; McWilliams, 2003; Pettigrew, 2004; Mert, 2005; Basal et al., 2009; Price, 2009; Rai, 2011; Hussein, Janat & Yakoub, 2011; Karademir, Karademir & Gençer, 2011; Cave, 2013). Fiber length is the fiber quality trait that is most affected by water stress. However, Ünlü et al. (2011) suggested that different irrigation doses did not have a significant effect on fiber quality measurements. Consistent with this study, McWilliams (2003), Başal et al. (2009), Rai (2011), Karademir, Karademir & Gençer (2011) and Price (2009) reported that water stress negatively affected fiber strength. In previous studies for fiber elongation, Karademir, Karademir & Gençer (2011) and Avsar (2021) reported that this trait was negatively affected, while Hussein, Janat & Yakoub (2011) reported that this trait was not affected by irrigation conditions.

The results of our PCA analysis are consistent with previous research on cotton genotypes (Saeed et al., 2015; Shabbir et al., 2016; Jamil, Khan & Ullah, 2020). PC1 and PC2 contributed the most to the overall variance in the experimental germplasm of the first four PCs, which is consistent with previous PCA results (Amna et al., 2013; Isong, Balu & Ramakrishnan, 2017; Zafar et al., 2022).

Assessing drought tolerance is complex due to the intricate mechanisms plants employ to cope with water stress. A multivariate approach is necessary for a more effective analysis of a plant’s response to well-watered conditions.

PCA, heatmap clustering and correlation analysis can reduce the number of correlated variables into a smaller set of representative variables known which in turn can be used to simplify numerous variables. Relevant indicators for drought tolerance have been identified and PCA, Heatmap and correlation analysis has been implemented to analyze and assess such resistance in rice, maize and wheat. Wen et al. (2017) and Kakar et al. (2019), Ghodrat & Bahrani (2022) and Zafar et al. (2022) has been used these analyses their studies.

Various statistical analyses were carried out under both full and limited irrigation conditions and the following conclusions were reached. As a result of cluster analysis, the parameter FS was found to be the most discriminative as it defined all colors from dark blue to dark red; as a result of correlation analysis, the highest correlation coefficient was found between FL and FU; and as a result of PCA, the parameters with the highest variability were FL and FU.

## Conclusion

According to this study, the relationship between genotype and water irrigation regimes significantly influences several cotton traits, including lint percentage (LP), boll weight (BW), fiber fineness (FF), fiber strength (FS), and fiber elongation (FE). Analyses revealed that certain lines, such as LTDS-117 and LTDS-116, exhibited superior values for boll weight (BW) and fiber strength (FS) across different irrigation conditions.

Cluster analysis grouped traits like boll weight and fiber strength together under both well-watered and stressed conditions. This suggests the importance of tailored breeding programs targeting specific traits in response to environmental conditions

The study recommends breeding for traits like yield, fiber fineness, fiber length, and fiber strength under deficit irrigation conditions. Conversely, for lint percentage, fiber uniformity, and fiber elongation, breeding under well-watered conditions is preferred.

Focusing on boll weight and fiber strength (FS) is crucial for successful selection in drought stress breeding programs, especially under optimal conditions. Conversely, fiber uniformity is deemed unsuitable as a selection criterion for drought stress due to consistent performance across irrigation conditions.

Pairwise discriminant analysis highlighted LTDS-128 and LTDS-116 as lines exhibiting the highest stress tolerance, showcasing their potential as drought-stress tolerant varieties.

The findings of this study provide strong support for the concept of implementing breeding programs for drought stress. Additionally, it has the potential to make a substantial contribution to the field of breeding studies, contributing not only to the confirmation of current studies but also to the development of a road map for further research in this area.

## Supplemental Information

10.7717/peerj.17584/supp-1 Supplemental Information 1 Deficit and well watered dataset

10.7717/peerj.17584/supp-2 Supplemental Information 2 Characteristics of variations used in breeding

## Additional Information and Declarations

### Data Availability

The following information was supplied regarding data availability: The raw data are available in the Supplemental File.
