# Supplementary material for: Unraveling the complexities of drought stress in cotton: a multifaceted analysis of selection criteria and breeding approaches
Source: PeerJ. 2024 Jun 24;12:e17584. doi: 10.7717/peerj.17584 (PMC11210482; doi:10.7717/peerj.17584)
Supplement: Supplemental Information 2 [file peerj-12-17584-s002.docx]

| **Carmen** | This variety was registered in Turkey in 2004. It is tolerant to Verticillium wilt and has high fiber quality. It is one of the preferred varieties in the Turkey region. |
| --- | --- |
| **STN-453** | It is one of the preferred varieties in the Southeast Anatolia Region in Turkey. |
| **Sahin-2000** | The Nazilli Cotton Research Institute (located in Aydın, Turkey) developed this variety to be tolerant to both water stress and wilt disease. |
| **GSN-12** | The GSN-12 cotton variety was developed in 2007 for its high yield performance by the Nazilli Cotton Research Institute in Aydın, Turkey. |
| **BA-119** | This variety was registered by Özbuğday Tohumculuk A.Ş. In 2007, İzmir, Turkey with its high-performance yield. |
| **Tamcot 22** | Longenberger et al. (2007) registered Tamcot 22 cultivar as drought tolerant variety. |
| **SJ-U86** | In 2006, Ulloa et al. (2009), registered this genotype in the United States as its high yield capacity and good fiber quality features, also suitable for cultivation under heat stress. |
| **DPL 90** | One of the cotton cultivars in the United States is known as it has high resilience to drought and heat stress (Weaver and Locy 2005). |
| **NIAB 999 and NIAB 111** | Iqbal et al. (2006) reported these varieties as drought-resistant cotton cultivars in Pakistan through the use of hybridization and mutation breeding techniques. |
| **EVA** | According to a study conducted in Greece (VolauDakis et al., 2002), this variety is considered highly resistant to drought. |
| **AZ 31** | Doron Nevo from Agridera in Israel, this cotton genotype has a tolerant potential to drought (According to a personal interview). |

**Supplementary Table 1:** **Characteristics of variations used in breeding**
